# Epidemiology of Sjögren’s Syndrome—from an Oral Perspective

**DOI:** 10.1007/s40496-016-0112-0

**Published:** 2016-09-02

**Authors:** Anne Isine Bolstad, Kathrine Skarstein

**Affiliations:** 1 Department of Clinical Dentistry, Faculty of Medicine and Dentistry, University of Bergen, Årstadveien 19, N-5009 Bergen, Norway; 2 Gade Laboratory for Pathology, Department of Clinical Medicine, Faculty of Medicine and Dentistry, University of Bergen, Bergen, Norway; 3 Department of Pathology, Haukeland University Hospital, N-5021 Bergen, Norway

**Keywords:** Sjögren’s syndrome, Xerostomia, Periodontitis, Interferon, Lymphotoxin, CXCL13

## Abstract

Oral symptoms are among the most distressing manifestations for patients with Sjögren’s syndrome (SS). The feeling of dry mouth is unpleasant, and hyposalivation may contribute to difficulty in speaking, chewing and swallowing and reduced quality of life. Reduced salivary flow increases the risk for dental caries and problems with prosthetic replacement. It seems that SS is not as frequently occurring as previously anticipated. Population-based prevalence studies on primary SS in Europe, conducted on large background populations and in accordance with the AECG criteria, reported of a prevalence of 1–9 cases per 10,000 people. This gives a combined prevalence of nearly 39/100,000 (~0.04 %). The cause of Sjögren’s syndrome is even now not fully understood, and the treatment of oral symptoms is still mostly palliative. Hopefully, useful information will appear from the new methods that are now available for genome wide association studies, epigenetics, DNA methylation studies, and proteomics. Similarly, this is anticipated for the immunological side of the story. The interferon signature, the interferon γ/interferon α mRNA ratio, and CXCL13 are among the proposed biomarkers of active disease. In this review, we provide an update on oral aspects of Sjögren’s syndrome with emphasis on the latest publications on these topics.

## Epidemiology of Sjögren’s Syndrome

Sjögren’s syndrome (SS) is a complex autoimmune disease characterized by a broad spectrum of clinical and serological manifestations. Patients with SS experience immune-mediated destruction of salivary and lacrimal glands leading to reduced lacrimal and salivary flow. Dry eyes and dry mouth together with fatigue are among the most common complaints [1, 2]. Extraglandular manifestations, such as involvement of the peripheral nervous, pulmonary, or gastrointestinal system, kidney, skin, myalgias, and arthralgias are frequently seen leading to reduced quality of life [3]. A range of autoantibodies can be present in Sjögren’s syndrome such as anti-SSA/Ro (Sjögren’s syndrome autoantigen A) and anti-SSB/La (Sjögren’s syndrome autoantigen B) antibodies, rheumatoid factor, cryoglobulins, and antinuclear antibodies. Patients with primary SS also have an increased risk of B cell lymphomas [4]. If the disease occurs together with another autoimmune disease such as for instance rheumatoid arthritis or systemic lupus erythematosus, its designation is secondary SS.

During the past decade, the 2002 revised American European Consensus Group (AECG) classification criteria have been widely used in studies of SS [5]. Recently, American College of Rheumatology (ACR) proposed a new set of criteria based on objective tests [6]. Great concordance was experienced between these two sets of criteria [7•]. However, a discrepancy between clinical diagnosis and criteria was experienced by both sets of criteria [8]. To assess the extraglandular systemic manifestations, the European League Against Rheumatism (EULAR) promoted and supported an international collaborative study (the EULAR-SS Task Force) aimed at developing consensual disease activity indexes in SS [3]. This resulted in two indexes, the EULAR SS Disease Activity Index (ESSDAI) and the EULAR Sjögren’s Syndrome Patient Reported Index (ESSPRI). The ESSDAI provides a standardized instrument for the homogeneous evaluation of systemic activity in clinical trials and daily practice, and includes organ-by-organ definitions. Hopefully, this will provide the basis for further development of evidence-based diagnostic and therapeutic guidelines [3]. The ESSPRI assesses the patient’s symptoms. Very recently, ClinESSDAI was developed, which is a clinical score without biological domain, meant as a tool for biological studies which provides an accurate evaluation of disease activity independent of B-cell biomarkers [9].

Numerous classification criteria for pSS have been proposed, but the most widely used are the AECG criteria. To accurately estimate the prevalence in Europe, we have to focus on robust studies using good methodology and large background population. To estimate the prevalence of pSS in Europe, Cornec and Chiche [10] appointed three population-based studies which had used the AECG classification criteria, which were based on large background populations, and which had used effective case-finding methods [11 12•, 13•]. A prevalence of 1–9 cases per 10,000 people was found, which gave a combined prevalence of nearly 39/100,000 (~0.04 %), which is far lower than previously reported (up to 6 %) [10]. In Europe, a disease is considered rare when it affects less than one person per 2000, and pSS may thus be regarded as a rare disease.

Table 1 gives an overview of some prevalence studies of pSS in Europe. Of note, although the Maldini et al.’s French study [13•] is based on a sample survey estimating population ≥ 15 years old, this study gives the lowest prevalence figure by using the AECG criteria [5]. The study by Kabasakal et al. [14] was based on a female population which may contribute to the higher prevalence. The estimate of point prevalence of pSS in both sexes was 0.15 %, and in the female population 0.29 % in the study by Trontzas and Andrianakos [15]. More data especially from non-European locations are warranted to definitively describe the epidemiological aspects of SS.

**Table 1:** Prevalence studies on primary Sjögren’s syndrome in Europe according to the AECG criteria

Author	Publication year	Country	Population size	Criteria	Prevalence %
					(95 % CI)
Trontzas & Andrianakos [15]	2005	Greece	8740	AECG	0.15 (0.09–0.21)
Alamanos et al. [11]	2006	Greece	488,435	AECG	0.09 (0.08–0.1)^b^
Kabasakal et al. [14]	2006	Turkey	831	AECG	0.72 (0.33–1.57)^a^
Birlik et al. [97]	2009	Turkey	2835	AECG	0.21 (0.03–0.29)
Anagnostopoulos et al. [98]	2010	Greece	3528	AECG	0.23 (0.22–0.75)
Göransson et al. [12•]	2011	Norway	852,342	AECG	0.05 (0.048–0.052)^b^
Maldini et al. [13•]	2014	France	1,172,482	AECG	0.01 (0.01–0.02)^b^

^a^The prevalence of pSS in the study of Kabasakal et al. was based on a female population only

^b^Population-based studies conducted on a large background population

## Dry Mouth and Saliva

Xerostomia refer to the subjective feeling of dry mouth and can present in people with normal saliva secretion. Hyposalivation can be objectively measured and typically is below 0.1 mL/min [16]. The condition can be either temporary or chronic and can be caused by a range of factors of which the most common is xerogenic medications. Other reasons may be radiation and chemotherapy for head and neck cancers, hormone disorders, infections, or systemic autoimmune diseases such as SS.

Patients with xerostomia suffer not only from reduced quantity of saliva but also a reduced quality [17]. Whole saliva consists of two main components, serous and mucous, in addition to hundreds of other substances such as a great diversity of minerals, antibodies, glycoproteins, bacteria, and complex mixes of proteins, lipids and ions a.o. Saliva has many important functions. Salivary mucins act as a lubricating agent and create a protective layer on teeth and mucosa. Mucins coat ingested food particles allowing them to be smoothly swallowed. The antibacterial, antifungal, and antiviral agents in saliva regulate the oral flora and help to prevent oral infections [18].

Prolonged dry mouth may result in functional alterations, such as difficulty in speaking, chewing, and swallowing, and in wearing dental prosthesis [19]. Other consequences are the increased plaque accumulation, increased risk of dental caries and erosions, and symptoms in the mucous membranes, lips and tongue, angular cheilitis and reduced quality of life. As the saliva volume decreases, the concentrations of IgA, lactoferrin, salivary proteins and peptides also are diminished, and the susceptibility to Candida infections increases. Comorbid medical conditions and medication use may have substantial impact on oral symptoms in individuals with SS [20].

## Dental Caries and Periodontal Disease

Reduced salivary flow and changes in saliva composition make patients at a greater risk for development of dental caries. The important buffer and remineralization capacities are reduced, as well as the protective effect against microorganisms.

A higher number of decayed, missed, and filled tooth surfaces (DMFS) and teeth (DMFT) has been demonstrated in SS patients compared to healthy controls [21, 22], and caries may emerge at more unusual localizations such as on incisal, labial and root surfaces [23]. DMFS has been found inversely correlated to salivary flow rates [21].

Patients with SS harbor higher numbers of cariogenic and acidophilic micro-organisms such as *Streptococcus mutans* and *Lactobacillus species* than healthy control individuals [21].

Compared with individuals with subjective sicca complaints [24], SS patients have a higher plaque index, gingival index, papillary bleeding index, and DMFT. Others found no significant differences in DMFT when comparing SS patients with patients with xerostomia or other immune diseases [25]. The difference in DMFT score has by some been ascribed to the missing tooth component, but often the reasons for extraction are not known or reported, if it has been conducted because of caries or attachment loss [21, 24]. However, SS does not seem to contribute to more periodontal disease than is found in healthy individuals, although the reports are somewhat conflicting [24–29]. It should be noted that the studies in general have included few patients.

## Dental Implants

Patients with SS often suffer from a high incidence of caries as a consequence of reduced saliva production. This may result in the need of prosthetic replacements. In cases with widespread loss of teeth, when there was not enough dental support for conventional fixed prosthesis, the only treatment available in the past was complete or partial dentures. Considering the problems with dry mouth, patients with SS have great difficulties with wearing partial dentures, and even worse, full dentures. In the absence of the lubricating effect of saliva, the oral mucosa is prone to sores and chafing from the dental prosthesis. The retention of full dentures in edentulous jaws becomes inadequate, which often leads to problems with speech and eating and impaired health-related quality of life. In view of this, dental implant rehabilitation as an alternative treatment is of special interest.

Implant survival rates of dental implants in healthy individuals over a 10-year period are high, from around 93–97 % [30, 31]. However, peri-implant mucositis are quite common, and peri-implantitis is a challenge, reported by some to range between 10 % at an implant level and 20 % at a patient level [31]. A history of periodontitis influences the success negatively, even if the patients have been treated. The type and severity of the disease also seem to be of importance for future events. Patients with aggressive periodontitis exhibit decreased implant success and survival rates when compared with chronic periodontitis patients, and if compared with a non-periodontitis group, they have a higher incidence of peri-implantitis and bone loss [31].

So what is the prognosis for dental implants in patients with SS? These patients present with mucosal dryness and experience reduced protection by saliva and a higher plaque index, which could mean a difference [24]. Despite this, as already mentioned, it seems that this group of patients does not have more periodontal disease than systemically healthy individuals. Will they experience more peri-implantitis?

The number of studies on dental implants in SS is scarce and even more limited with regard to follow-up time. There are a few publications based on single case reports (for review see [32]) . As an example, Binon et al. [33] presented a case report with mandibular osseointegrated implants with a fixed complete denture remaining stable and functional during 13 years follow-up. Isidor et al. [34] reported of 84 % success rate after 4-year follow-up of 54 implants in 8 patients, while Payne et al. [35] achieved 88.4 % success rate of 26 implants placed in three patients but with only 2-year follow-up. A more recent publication examining 50 patients with SS found a prevalence of peri-implantitis of 14 % of the patients (11 % of the implants), which is similar to what is reported for systemically healthy individuals [36]. With a median follow-up of 46 months, the implant survival was 97 %; of the 142 implants inserted, four implants in two patients were lost. They also reported that oral functioning correlated negatively with oral dryness and chewing ability in these patients. These results were in line with a patient-reported investigation on outcomes of dental implants in 32 patients with SS. A total of 5 of 104 (4.8 %) implants had to be removed over a mean period of 4.9 years in the patients [37]. In summary, based on the available studies, implant survival rates in patients with SS seem to be comparable to those in systemically healthy individuals [38].

## Pathogenesis

### Salivary Gland Biopsy—Diagnostic Tool

In experienced hands, minor salivary gland biopsy is a well-tolerated procedure associated with a low complication rate [39]. While some patients are willing to have three or even four biopsies removed for the purposes of follow up and research, there is an ethical limit on the number of consecutive lip biopsies.

Salivary gland involvement has a central role when assessing the development and pathogenesis of SS. Notably, SS histopathology is strongly associated with autoantibodies but only correlates weakly with xerostomia in SS patients. The diagnostic role of salivary gland histology still remains widely accepted and a central part of both AECG and the new proposed classification criteria endorsed by the ACR in 2012 [6].

The autoantibodies Ro/SSA and La/SSB are valuable diagnostic tools, they appear early in the disease, persist, and correlate with focus scores. Histological focus scoring has been employed to describe salivary gland involvement in SS, where a positive biopsy with mononuclear cell infiltrates comprising of ≥50 mononuclear cells per 4 mm^2^ results in a positive score value of 1–12 according to numbers of foci seen.

Few studies have assessed other histopathological features in the salivary gland environment and their possible associations with diagnosis and stage of disease. The destruction of salivary gland tissue in SS is commonly accompanied by the development of adipose tissue and fibrosis where adipocytes can occupy a large part of the gland. A recent study [40] highlights the possibility that there could be a relationship between the disease activity and adipose tissue replacement in the gland, and that fat replacement could be a helpful support in the diagnostic evaluation of the glandular tissue. Furthermore, a possible active role of adipocytes in the immune reactions in the glandular environment has been suggested [40, 41]. During the last years, novel diagnostic tools have been investigated. These would include salivary gland ultrasound imaging where parenchymal inhomogeneity appears to be the method with most promising results [42]. Even if its role in the early stages of disease is debated, it is worthy of note that, when used in association with traditional tests, ultrasound improves the diagnostic sensitivity of the AECG [43–46].

Proteomic biomarker profiles of unstimulated whole saliva from SS patients have been investigated for potential as a tool for patient subclassification [47]. Further studies will be necessary to determine the utility of such an approach. To facilitate the evaluation of treatment efficacy in clinical trials and to select subgroup of patients for personalized treatment, the availability of new prognostic markers is needed.

### Immunopathology—Cellular Populations

The classic glandular lesion is composed of a lymphoid infiltrate of T and B lymphocytes, whose distribution may vary according to disease severity. Macrophages, plasma cells, NK cells, and dendritic cells are also present in varying degree [48, 49]. A great effort has been made to in deeply characterizing the role of different T cell subsets in pSS. In pSS patients, the specific T helper subset, Th 17 cells, mainly defined by secretion of cytokine IL-17, has been found in elevated numbers both in the periphery and also present in the salivary gland tissue [50, 51]. The follicular T helper cells are another subset derived from naïve lymphocytes under the stimulus of IL-12 secreted by dendritic cells, and these cells are involved in the crosstalk between T and B cells. It has been indicated that these cells participate in the pathogenesis of pSS by promoting B-cell maturation [52].

B cell hyperactivity represents a key hallmark in the pathogenesis of SS and hypergammaglobulinemia, autoantibody production and alterations of B cell subpopulations are distinctive features of pSS patients. Patients with pSS present up to 16 times increased risk for developing non-Hodgkins lymphoma (NHL) [4] and mucosa associated lymphoid tissue (MALT) lymphoma in the salivary glands and gastrointestinal tract compared to healthy individuals. Lymphoid organization in the form of germinal center (GC)-like structures has been identified in the salivary glands of a subgroup of SS patients [53]. Notably, the identification of germinal center-like structures has been suggested to be a possible predictor of the development of lymphoma since, based on results from a study from Theander et al., the majority of the GC+ patients developed lymphoma later on [54]. This novel finding may allow identification of high-risk patients for repeated lymphoma screening and selection of candidates for advanced B-cell directed biological treatment.

Negative status for anti-Ro/SSA and/or anti-La/SSB is suggested to be a protective factor for evolution toward lymphoma in these patients [55]. However, the development of lymphomas in pSS is not confined only to serologically positive patients for anti-Ro and anti-La. Accordingly, the rheumatologists are encouraged to include the minor salivary gland biopsy in the routine work-up [54]. CD4+ T lymphocytopenia is an additional strong risk factor for developing lymphoma [4]. Lymphomas often develop in salivary glands of SS patients where the disease is active. Nocturne and Mariette recently launched a “2014 proposed scenario for the pathophysiology of pSS-associated lymphoma” [56]. They envisioned that immune complexes with antibodies against specific antigens such as SSA/Ro and SSB/La or others, continuously stimulate autoimmune B cells containing rheumatoid factor activity. Furthermore, that defects in control of NF-kB activation accentuate B cell over-activation and promote survival of B cells and oncogenic mutations.

### Genetics

The HLA carries the major genetic influence on susceptibility to autoimmune diseases, as it is an important key in antigen presentation and immune response. A meta-analysis found DRB1*03:01, DQA1*05:01, DQB1*02.01, and DRB1*03 to be risk factors for pSS while DQA1*02:01, DQA1*03:01, and DQB1*05:01 alleles were protective [57]. DRB1*03-DQB1*02 was the significant risk haplotype associated with pSS on a worldwide level in that study. This is in line with our previous study where we also found components of the DRB1*03-DQB1*02-DQA1*0501 haplotype as the strongest contributors to the formation of an anti-Ro/La response in a study of pSS Caucasions [58].

Several non-HLA regions have been implicated in pSS. Gene expression microarray studies on labial salivary glands and peripheral blood showed dysregulation of type I interferon-inducible genes [59, 60]. Two GWAS studies have recently been performed on pSS, one on European descents, and one on Chinese descents [61••, 62••]. Among SS-associated non-HLA genes discovered by GWAS, we find *STAT4* and *IRF5* encoding transcription factors, *BLK* coding for B cell kinase, as well as genes encoding the IL-12A cytokine, and interestingly, genes involved in NF-kB signaling and the CXCR5 chemokine production [63]. CXCR5 is receptor of CXCL13, which directs B cells to lymphoid follicles [64]. Mice deficient of Cxcl13 or its receptor Cxcr5 fail to form these structures [64, 65]. In addition to the interferon signature appearing from the microarray gene expression profiling of minor salivary glands of primary SS patients and healthy controls, particularly two other interesting observations were made by Hjelmervik et al. [59]. Firstly, CXCL13 which directs B cells chemotaxis was differently expressed in patients and controls. *CXCL13* were among the genes that were expressed in 9 of 10 patients with pSS, whereas it was only expressed in 1 of 10 healthy individuals [59]. This is supported by a recent study by Kramer et al. who found CXCL13 to be elevated in serum and saliva of SS patient and in mice models [66]. Secondly, we found lymphotoxin-β (*LTB*) to be among the most highly expressed genes in inflamed salivary glands of pSS patients. Lymphotoxin-β receptor (LTβR) signaling is crucial for the formation of lymphoid tissue, and LTβ can activate NF-kB pathways that promote inflammation [67–69]. When we in a later experiment blocked the Ltβr in animal models of SS, there was an increase in salivary secretion and a reduction of inflammation in the glands [70]. We experienced an amelioration of SS after neutralization of Ltβr signaling. A congenic mouse model was also developed that differed genotypically from the control mice only in two non-MHC loci, which were sufficient for the congenic mice to develop sialadenitis spontaneously [71]. *Cxcl13* and *Ltβ* were among the genes that were found differentially expressed in salivary glands of the NOD congenic mice compared to control mice. Furthermore, Ltβ blockade also reduced Cxcl13 in lacrimal glands of a NOD model of SS improving the corneal integrity [72, 73]. Recently, early BAFF receptor blockade was shown to mitigate murine SS [74•]. Concomitant targeting of *CXCL13* and *BAFF* receptors prevented salivary hypofunction [74•]. CXCL13 has been proposed as a biomarker for SS and a possible therapeutic target [66].

Epigenetic factors such as altered patterns of DNA methylation have been implicated in models of autoimmune disease. Recent studies have reported epigenetic alterations such as changes in DNA methylation, histone modification, micro-RNA expression, and have found defective DNA methylation to be associated with SSB gene expression and lymphocyte infiltration in pSS [75–77]. Just recently, a genome-wide DNA methylation study on human labial salivary glands of SS was presented [78]. Several genes and pathways previously thought to be involved in disease-related processes as well as a number of new candidates were discovered. Interestingly, a correlation was recognized between DNA methylation and a set of genes previously found highly differentially expressed in pSS and healthy salivary glands [59, 78]. Furthermore, genome-wide DNA methylation profiles showed prominent hypomethylation of interferon-regulated genes in whole blood and CD19+ cells in pSS [79•].

As mentioned, increased expression of type I interferon-regulated genes have been demonstrated in autoimmune diseases [80]. An interferon signature was demonstrated in sera and minor salivary glands of pSS [59, 81], and in CD14 monocytes of pSS patients it was found associated with disease activity and higher B cell activation factor (BAFF) gene expression [82]. *CXCL10* is one of the most strongly upregulated type I interferon-regulated genes and was found upregulated in salivary glands of pSS patients [59, 80]. A causal relationship between type I interferon production and development of autoimmune disease has been suggested, and as a biomarker of active disease, the interferon signature is an interesting target for research aiming at new treatment possibilities [80]. The contribution of type I and type II interferon signatures to SS pathogenesis and lymphomagenesis was recently investigated, and interferon γ/interferon α mRNA ratio was proposed as a novel biomarker for prediction of in situ lymphoma development in SS [83].

Type I interferons are involved in innate immune response against viral infection and help to regulate the activity of the immune system. A link between the LTβR and interferon pathways and mouse models has pointed to the pathogenic role of the lymphotoxin and interferon I pathways in human autoimmune diseases [69]. The lymphotoxin pathway has a role in orchestrating the development of homeostasis of lymph nodes through regulation of homeostatic chemokines, and the LTβR signaling is essential in differentiating stromal cells and macrophages in lymphoid organs to produce interferon I in response to virus infection. A possible potential of the lymphotoxin network as a tool for treatment of autoimmune diseases has been suggested [69].

Virus has been suggested as one of the environmental factors that could trigger SS and be involved in SS pathogenesis, and the observation that infection can precipitate autoimmune abnormality is not new [84]. A number of candidates have been suspected to trigger such a disease, such as human herpes virus 6 (HHV6), cytomegalovirus (CMV), Epstein-Barr virus (EBV), hepatitis C virus (HCV), human T lymphotropic virus type 1 (HTLV-I), and human immunodeficiency viruses (HIV) [85]. However, the connection is not clear, and more studies are justified.

Autoimmune diseases aggregate in families. In a population-based family study of 105 Taiwan patients with SS who had an affected first-degree relative, Kuo et al. [86] found a relative risk (RR) for SS in siblings of patients with SS to be 18.99, 11.31 in offspring, and 12.46 in parents. In first-degree relatives of SS patients the RR were 6.25 for having systemic lupus erythematosus, 3.38 for multiple sclerosis and 2.95 for rheumatoid arthritis [86].

## Treatment

### Intervention for Dry Mouth

Often dry mouth cannot be cured, but there are ways to decrease the dry mouth symptoms and improve the feeling of dry mouth, at least temporarily. Not at least, it is mandatory to implement measures to reduce caries risk in patients with hyposalivation.

Some patients feel that palliative treatment with salivary substitutes, chewing gum and sugarfree lozenges help to some extent, but the effect is short-lived. The symptomatic and supportive treatment of dry mouth can be either local or systemic [87]. Sugar-free gum, mint, and lozenges are recommended for salivary stimulation. Effort has been made to produce artificial saliva which mimics the normal and protective effects of saliva, with addition of a.o. remineralizising and antimicrobial agents. The effect of topical treatments in reducing symptoms of dry mouth was recently the topic of a Cochrane database systemic review [16]. Sprays, lozenges, mouthrinses, gels, oils, chewing gum, and toothpastes were evaluated in this review; however, there was no strong evidence that any topical treatment was effective for relieving the sensation of dry mouth. An oxygenated glycerol triester saliva substitute spray was more effective than a water-based electrolyte spray. Chewing gum increased saliva production, but there was no evidence that gum was better or worse than saliva substitutes.

A very important supplement in patients with hyposalivation is the use of fluoride to decrease tooth decay. Daily use of fluoridated dentifrice and fluoride rinse is necessary remineralization options. Fluoride gel in mouth guard is an alternative in serious cases. Toothpaste with sodium lauryl sulfate and acidic products and those containing sugar should be avoided [88, 89].

Prophylaxis, more frequent dental visits with professional cleaning, oral hygiene instructions and motivation should be accomplished, including application of topical fluoride gel when indicated. More detailed treatment strategies for xerostomia, and overview of salivary stimulators, oral moisturizers, and salivary substitutes can be found in recent published comprehensive reviews [87, 90].

SS patients are prone to fungal infection. Antifungal rinses or lozenges are available for treatment of oral candidiasis. If patients have removable prostheses, the prostheses may be soaked in for instance a chlorhexidine solution. One must be aware of the discoloration this procedure could cause.

### Physiological and Pharmacological Stimulation and Gene Therapy

There is insufficient evidence to determine the effects of electrostimulation devices on dry mouth symptoms or saliva production in SS [91]. Similarly, any better effect of acupuncture compared to placebo has been difficult to prove. Insufficient number of patients makes it difficult to conclude.

The muscarinic receptor agonists pilocarpine and civemeline may have good effect in stimulating to increased salivary secretion [92]. Pilocarpine was associated with improvements in dry mouth of 61–70 vs. 24–31 % in placebo, whereas civemeline was associated with improvement in dry mouth of 66–76 vs. 35–37 % in placebo [88]. Some regard pilocarpine as the best-performing sialagogues drug for SS [93].

There has been an interest in salivary glands as delivery organs for gene therapy. The glands have several advantages in that respect, being easily accessible, well encapsulated, and non-critical for life organs with extraordinary secretory abilities, and producing proteins for transport in exocrine and endocrine directions [94]. Salivary gland gene transfer is easily accomplished and can be done in a non-invasive manner [95]. In 2003, the first publication was released showing transfer of a gene encoding immunomodulatory protein resulting in improved salivary gland function and morphology in a SS mouse model [96]. To be used for treatment in SS, however, gene therapeutics seems yet far away, also because of our lack of complete understanding of the disease process.

## Summary

In this review, we have focused on the most recent literature on SS epidemiology from an oral perspective, including some references giving necessary background for the discussion of the topics. To be updated on the most recent literature regarding pathogenesis, genetic aspects, clinical and basic features, and treatment of SS in general, we refer to the review from Ferro et al. [7•].

It is clear that the SS may entail serious oral problems of importance for quality of life in addition to impaired systemic health condition. More research is needed to understand the disease pathogenesis and to identify the initiating factor (s) of SS, to be able to perform early diagnostics and improve the treatment of the disease.
